# Data related to the manufacturing and mechanical performance of 3D-printed metal honeycombs

**DOI:** 10.1016/j.dib.2022.108857

**Published:** 2022-12-25

**Authors:** Shahriar Afkhami, Mohsen Amraei, Ilkka Poutiainen, Leroy Gardner, Heidi Piili, M. Ahmer Wadee, Antti Salminen, Timo Björk

**Affiliations:** a Laboratory of Steel Structures, LUT University, Lappeenranta 53850, Finland; b Department of Mechanical and Materials Engineering, University of Turku, Turku 20520, Finland; c Laboratory of Laser Materials Processing and Additive Manufacturing, LUT University, Lappeenranta 53850, Finland; d Department of Civil and Environmental Engineering, Imperial College London, London, UK

**Keywords:** Laser-based powder bed fusion, Digital manufacturing, Steel, Honeycomb structures, Hollow structures, Strain rate, Compression load, Impact load

## Abstract

The data available in this article include 3D mechanical designs used for the computer-aided fabrication of metal honeycombs produced by additive manufacturing and studied in [1]. In addition, the force-displacement data utilized to evaluate the mechanical performance of the metal used in this study are available via the digital image correlation technique. Further, the surface features obtained using 3D scanning microscopy of the fabricated parts are available as raw files and processed data. Finally, the impact test data are presented as high-frame-rate videos showing the time-displacement numerical values. This information has been provided in this data article to complement the related research, serve as a guide for future studies, and ensure the data's repeatability and reliability of the related research paper. The research article [1] investigates the mechanical performance and failure mechanism of additively manufactured metallic honeycombs under various scenarios, from quasi-static to dynamic loading. It also investigates the design optimization of these energy-absorbing hollow structures by comparing hollow structures made of three distinct novel cell designs (triangular, diamond-shaped, and diamond-shaped with curved walls) with traditional honeycombs made of hexagonal cells.

**Specifications Table**

**Table:** 

Subject	Engineering
Specific subject area	Mechanical Engineering
Type of data	Images Graphs Figures Videos Tables
How the data were acquired	The microstructural images were obtained using a Hitachi SU3500 scanning electron microscope (SEM). Curves in Fig. 2 and their corresponding videos were produced using an ARAMIS digital image correlation (DIC) system in conjunction with a Galdabini Quasar 600 universal testing machine. The illustrations in Fig. 3 and their corresponding STL files are made using SolidWorks 2021. Surface and topological features were captured via a KEYENCE VE-3200 microscope. The load-displacement data from the compression tests were obtained via two in-house test rigs with 200 kN and 5000 kN loading capacities. Finally, the impact test videos were recorded via a Phantom VEO 710L high-speed camera.
Data format	Raw Analyzed Filtered
Description of data collection	Specimens were manufactured by an EOS M290 additive manufacturing system using the parameters noted in [1]. Images from SEM were acquired by mounting a cuboidal specimen in epoxy resin and studying it under the microscope. A scanning voltage of 15 kV was used for taking the images through the secondary electron detector. The tensile test was carried out per ASTM E08[2] at room temperature. Compression tests and impact tests were also carried out at room temperature.
Data source location	• Institution: LUT University • City/Town/Region: Lappeenranta/ South Karelia • Country: Finland
Data accessibility	Repository name: Additively manufactured metal honeycombs Data identification number: 10.17632/8bxkdw4nfw.1 Direct URL to data: https://doi.org/10.17632/8bxkdw4nfw.1
Related research article	S. Afkhami, M. Amraei, L. Gardner, H. Piili, M. A. Wadee, A. Salminen, T. Björk, Mechanical performance and design optimisation of metal honeycombs fabricated by laser powder bed fusion, Thin. Wall. Struct. 180 (2022) 109864. https://doi.org/10.1016/j.tws.2022.109864

## Value of the Data

**Fig. 1:**
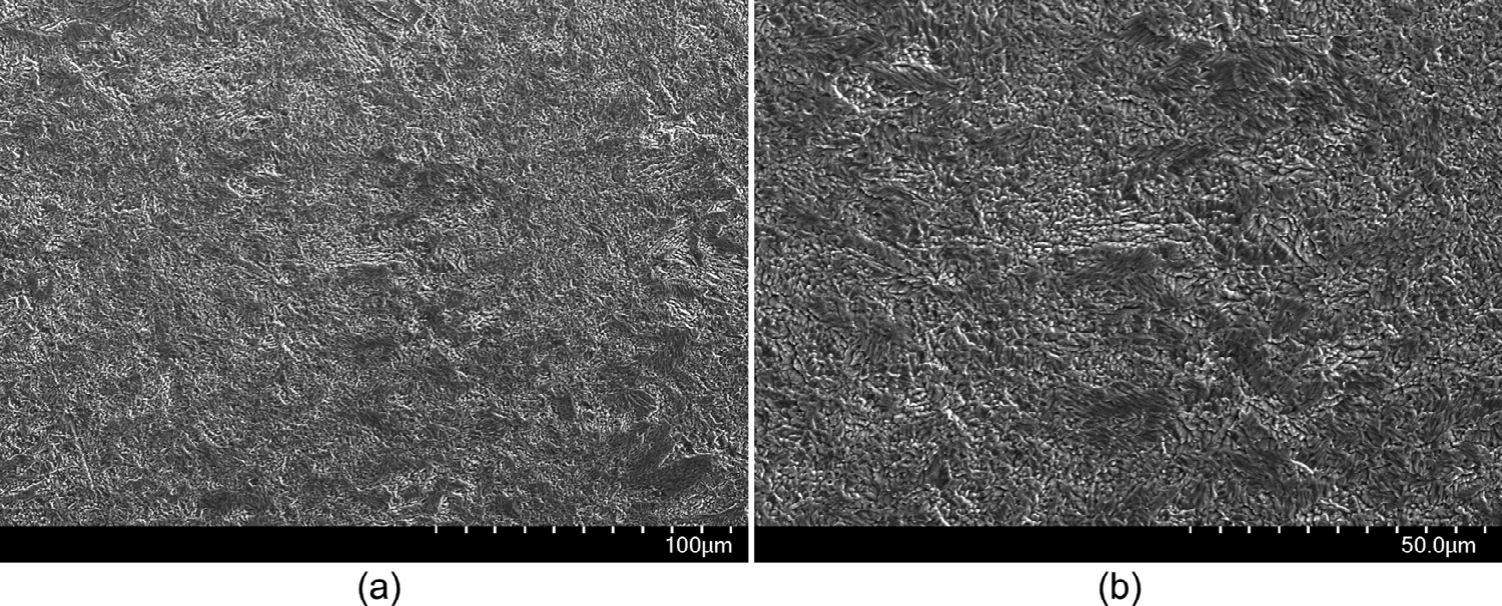
SEM micrographs of the manufactured material, stainless tool steel CX, processed via laser-based powder bed fusion (L-PBF CX): (a) 500X and (b) 1000X.

**Fig. 2:**
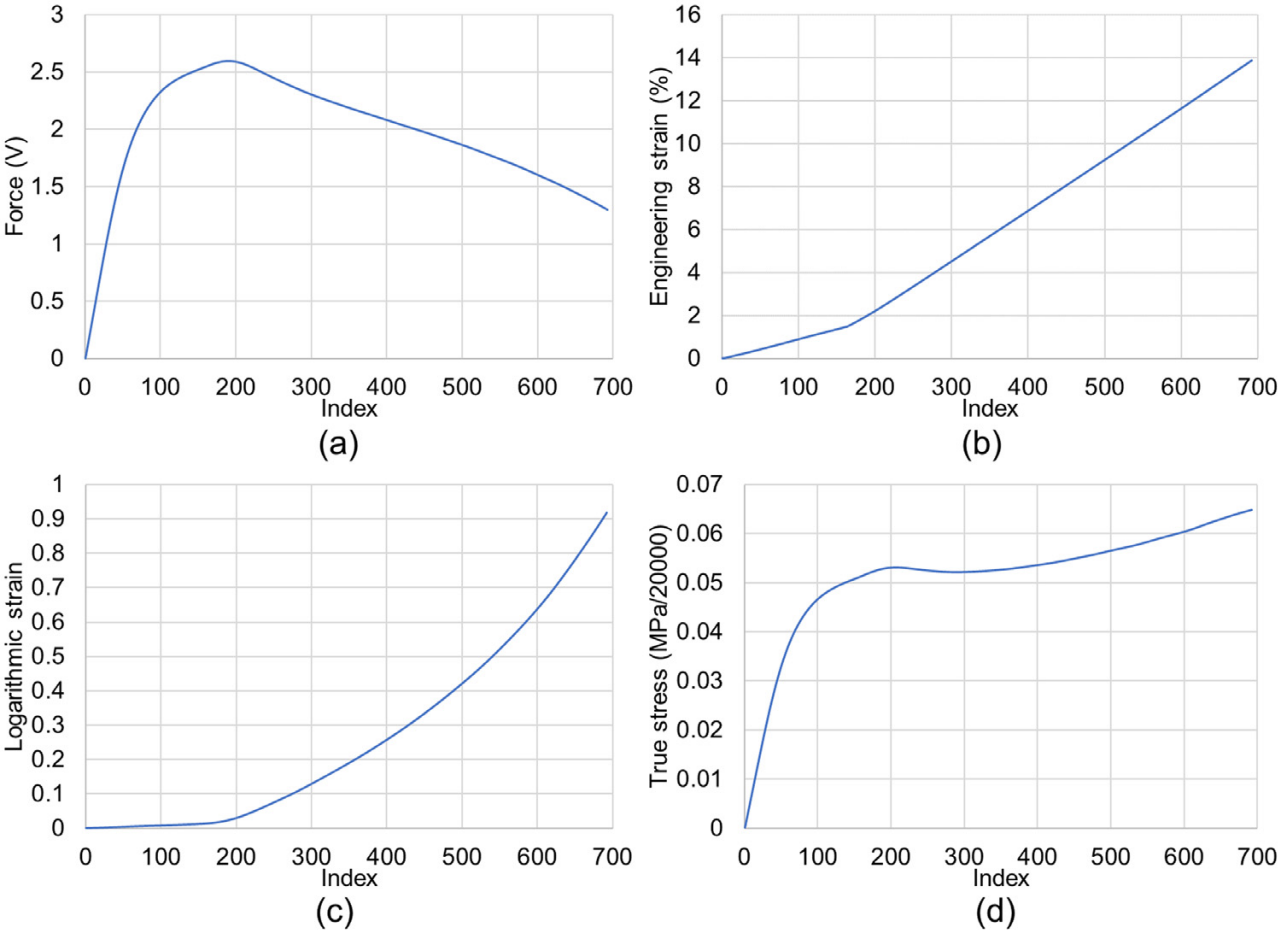
Data achieved via the digital image correlation technique while performing the quasi-static tensile test on a standardized specimen made of L-PBF CX.

**Fig. 3:**
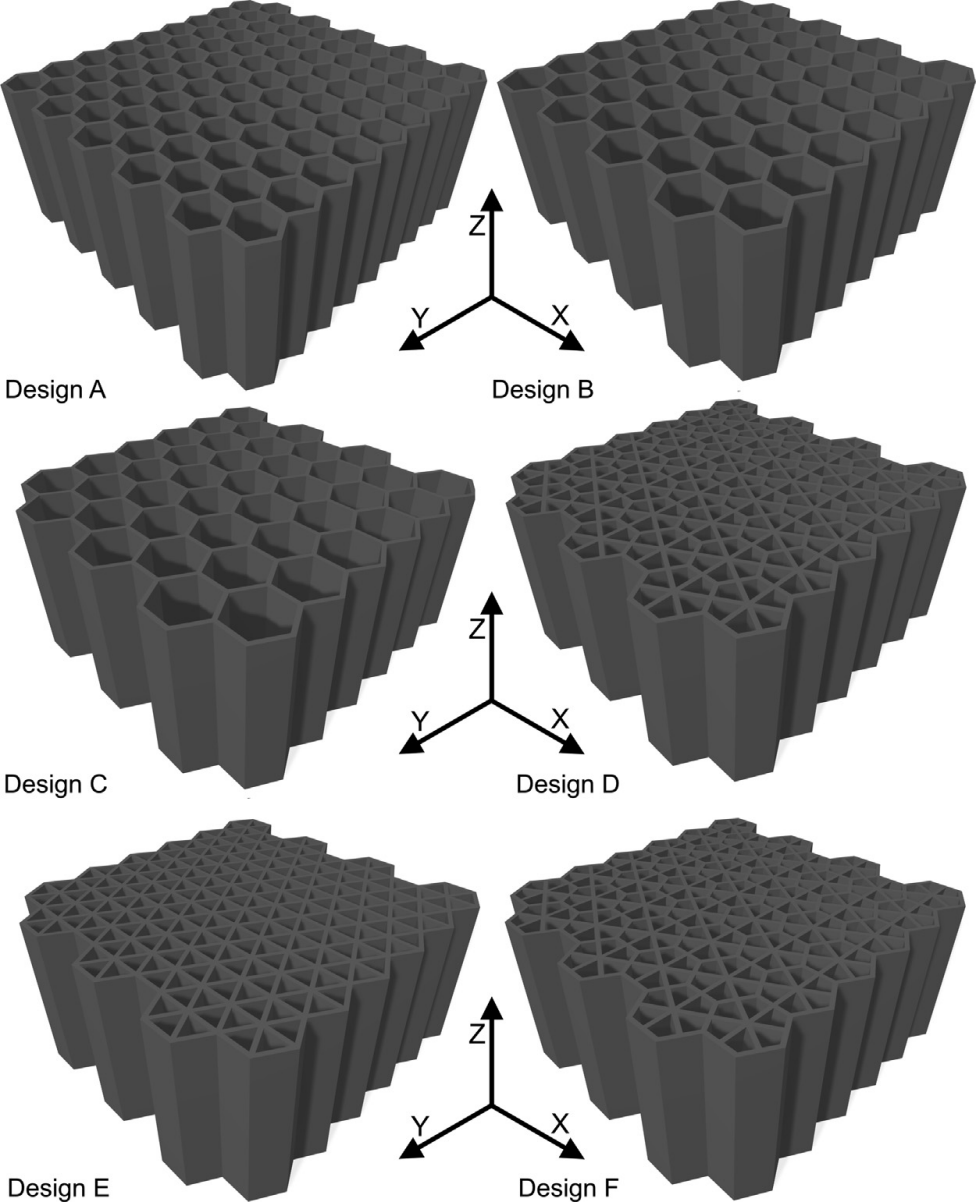
3D views of the designed samples from their corresponding STL files.

**Fig. 4:**
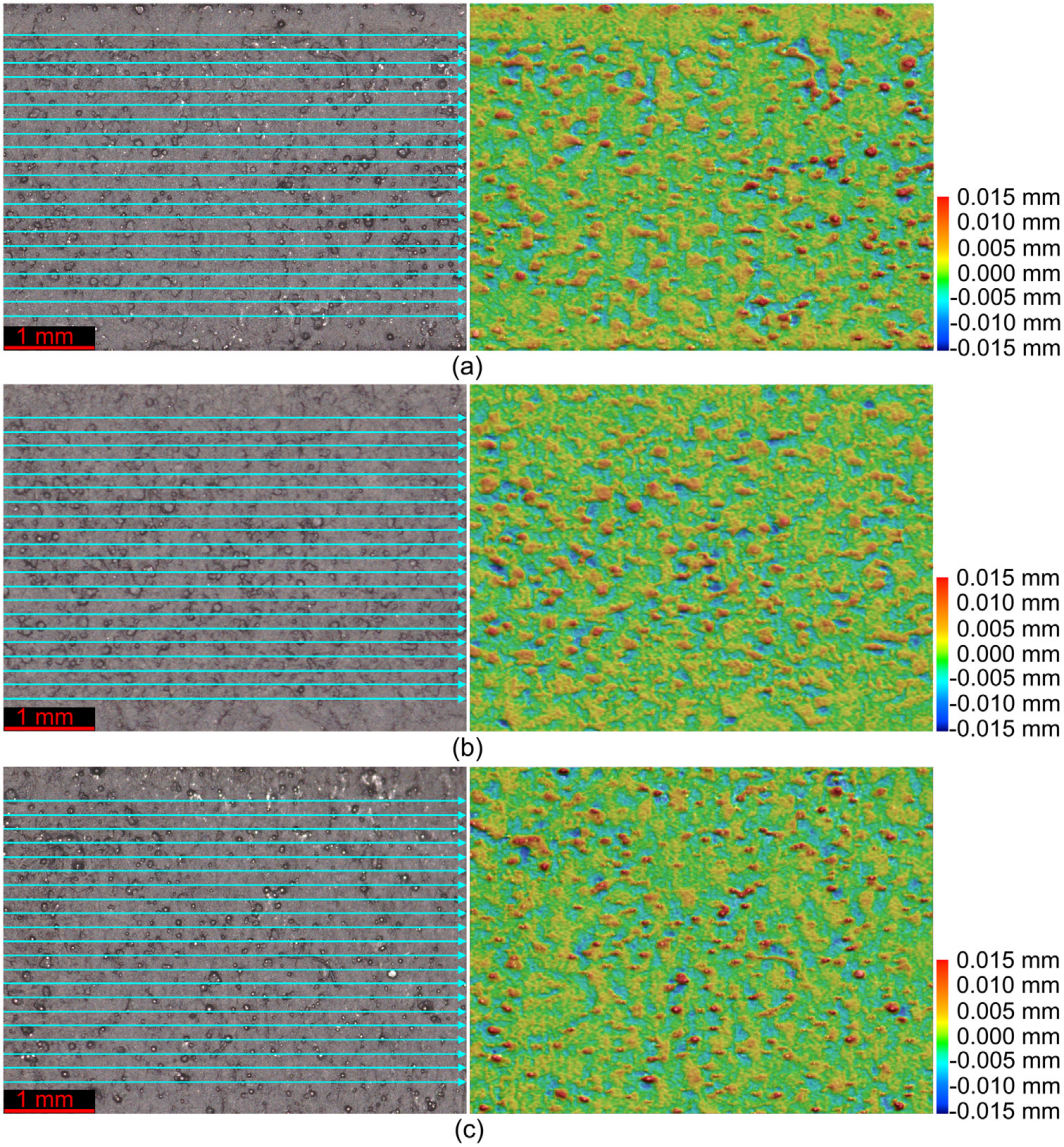
(left) Scanned lines and (right) topological features of the probed surfaces from (a) design A, (b) design B, and (c) design C.

• Microstructural images in Fig. 1 provide insight into the microstructural characteristics of the metal used to manufacture the samples.
• The data in Fig. 2, and their corresponding videos in the data repository, can be used to calibrate the material behavior for finite element (FE) models; the combination of this data set and the microstructural features from Fig. 1 can be helpful to study the correlations between the microstructural features and mechanical properties of additively manufactured metals.
• The designs in Fig. 3 and their corresponding STL files in the data repository can be used to fabricate specimens with the same features as the ones used in this study. Further, the STL files can be utilized to extract any geometrical details required for future research and help achieve the most repeatability from the current study and its associated research article [1].
• Surface and topological data in Fig. 4 can help researchers study the correlations between the design type, fabrication parameters, and surface quality of hollow structures fabricated by additive manufacturing methods, especially the laser-based powder bed fusion technique (L-PBF).
• Force-displacement data from the compression tests and their corresponding videos in the data repository can be used as benchmarks for FE models in future studies to ensure such models simulate the mechanical response of these honeycombs up to their final failures accurately.
• Displacement-time data can be extracted from the videos of the impact tests in the data repository to help researchers with modelling impact loading scenarios applied on hollow structures, such as the honeycombs used in the current study and its corresponding research article [1].

## 1 Objective

The dataset is generated to evaluate the capability of laser-based powder bed fusion as an additive manufacturing technique to fabricate metal honeycombs layer-by-layer and study the mechanical performance of such honeycombs under various loading scenarios. The force-displacement data, either for the compression or impact tests, are made comprehensively available in this data article since they were partly available in the related research paper due to length limitations. Further, visual data and videos of force-displacement-time sequences recorded during the mechanical tests are available in the data repository to help researchers better understand how these hollow structures behave under different loading scenarios.

## 2 Data Description

The microstructural features in Fig. 1 show the microstructure of stainless tool steel CX processed via laser-based powder bed fusion (L-PBF CX) in its as-built (not heat treated or post-processed) condition. The raw images directly taken from the SEM are available in the data repository as CX_500XSEM.tif and CX_1000XSEM.tif for Fig. 1(a) and 1(b), respectively.

Fig. 2 presents the local (true) and global (engineering) stress-strain-force-time data achieved through the quasi-static tensile test of L-PBF CX material. Each force signal and time index in this figure represents 20000 N force and 0.25 s time intervals, respectively. The raw data from the DIC system are available in the data repository as CX_Tensile.xlsx. Further, the tensile test progress was recorded via the DIC system, and the videos are available in the data repository as CX_Tensile_1.mp4 for force and engineering stress values and CX_Tensile_2.mp4 for true stress and logarithmic strain values. Finally, the calibration parameters of the DIC system are available in DIC_calibration.pdf from the data repository.

The illustrations of the specimens in Fig. 3 are made based on the STL files in the data repository: Design_A.stl, Design_B.stl, Design_C.stl, Design_E.stl, and Design_F.stl for the sample designs A, B, C, D, E, and F, respectively. These STL files were also directly used to fabricate the actual specimens via additive manufacturing.

Surface images and topological features in Fig. 4 are based on the raw data available in A.zon, B.zon, and C.zon from the data repository for Fig. 4(a), (b), and (c), respectively. The numerical data from these raw files are also available in Table 1.

**Fig. 5:**
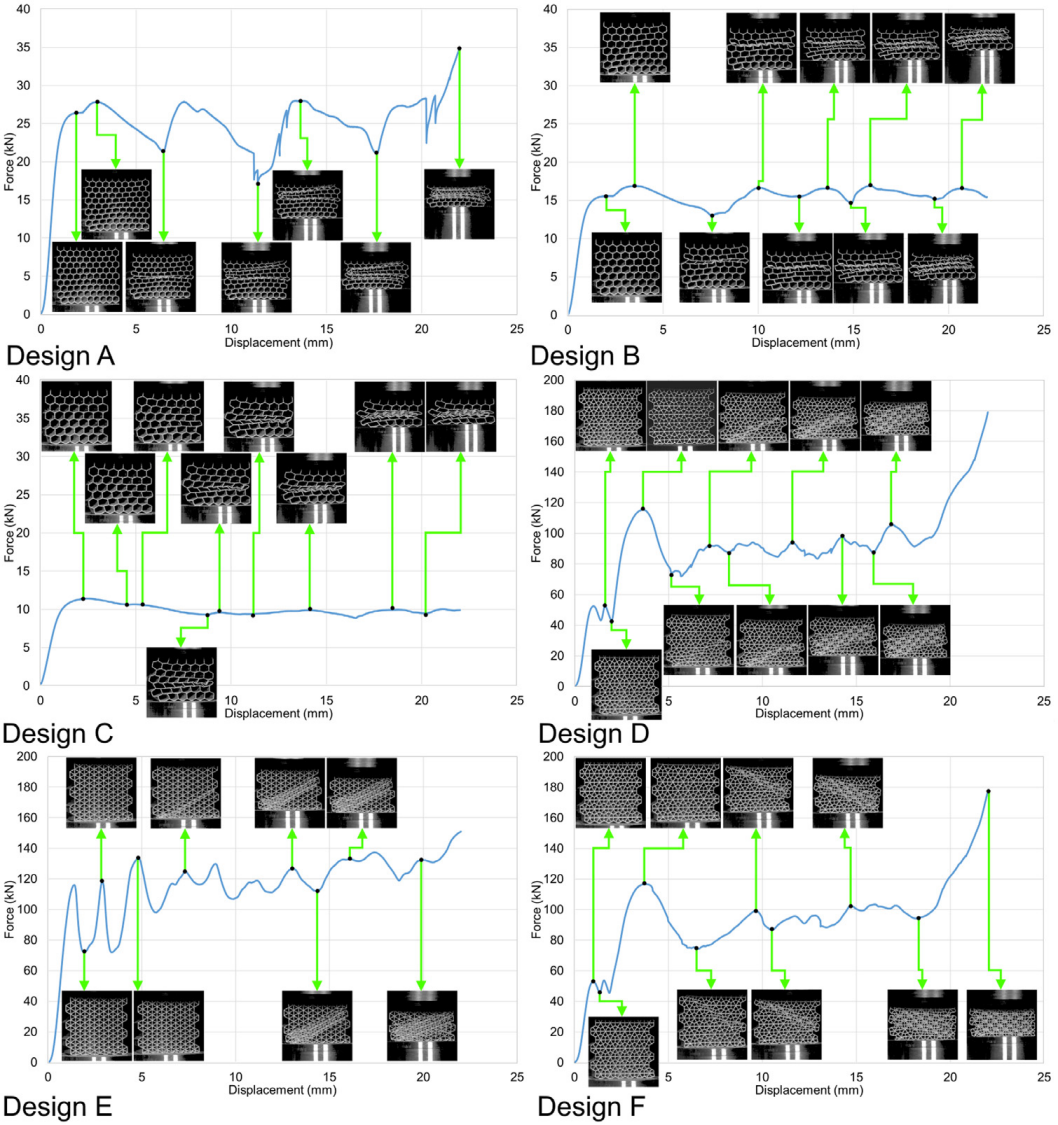
Force-displacement data of the specimens loaded along the X direction under the deformation rate of 0.1 mm/s.

**Fig. 6:**
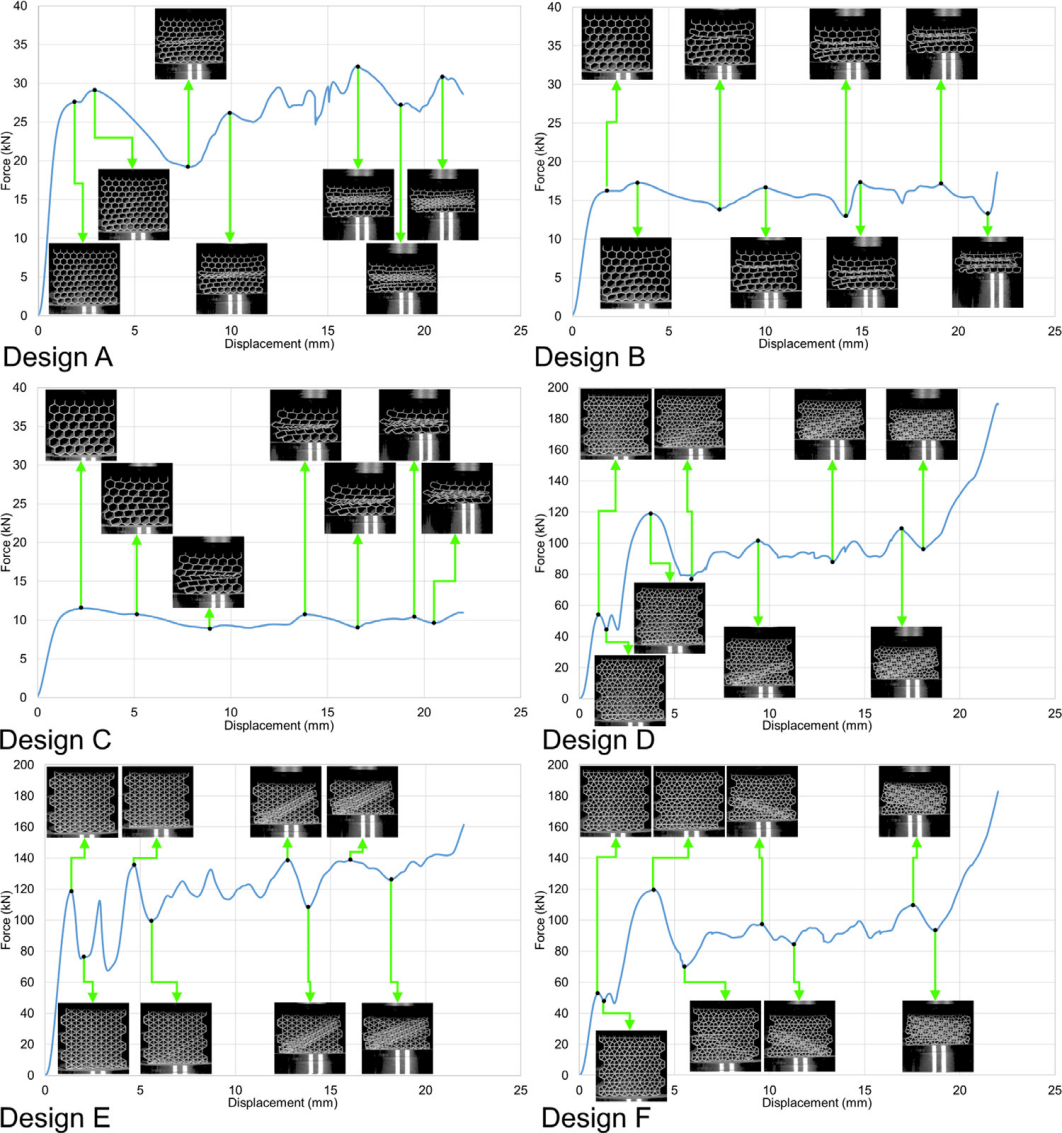
Force-displacement data of the specimens loaded along the X direction under the deformation rate of 1.8 mm/s.

**Fig. 7:**
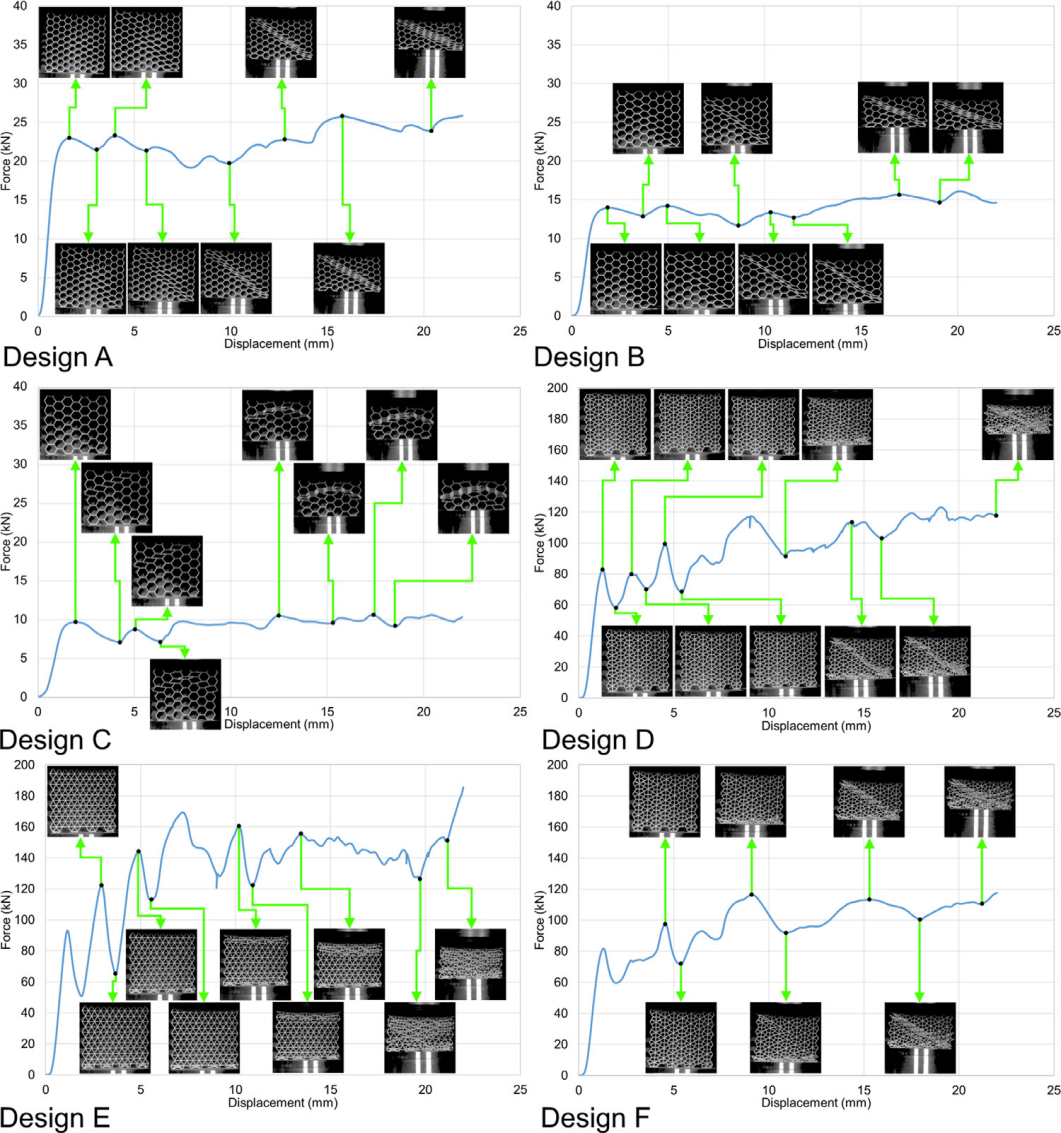
Force-displacement data of the specimens loaded along the Y direction under the deformation rate of 0.1 mm/s.

**Fig. 8:**
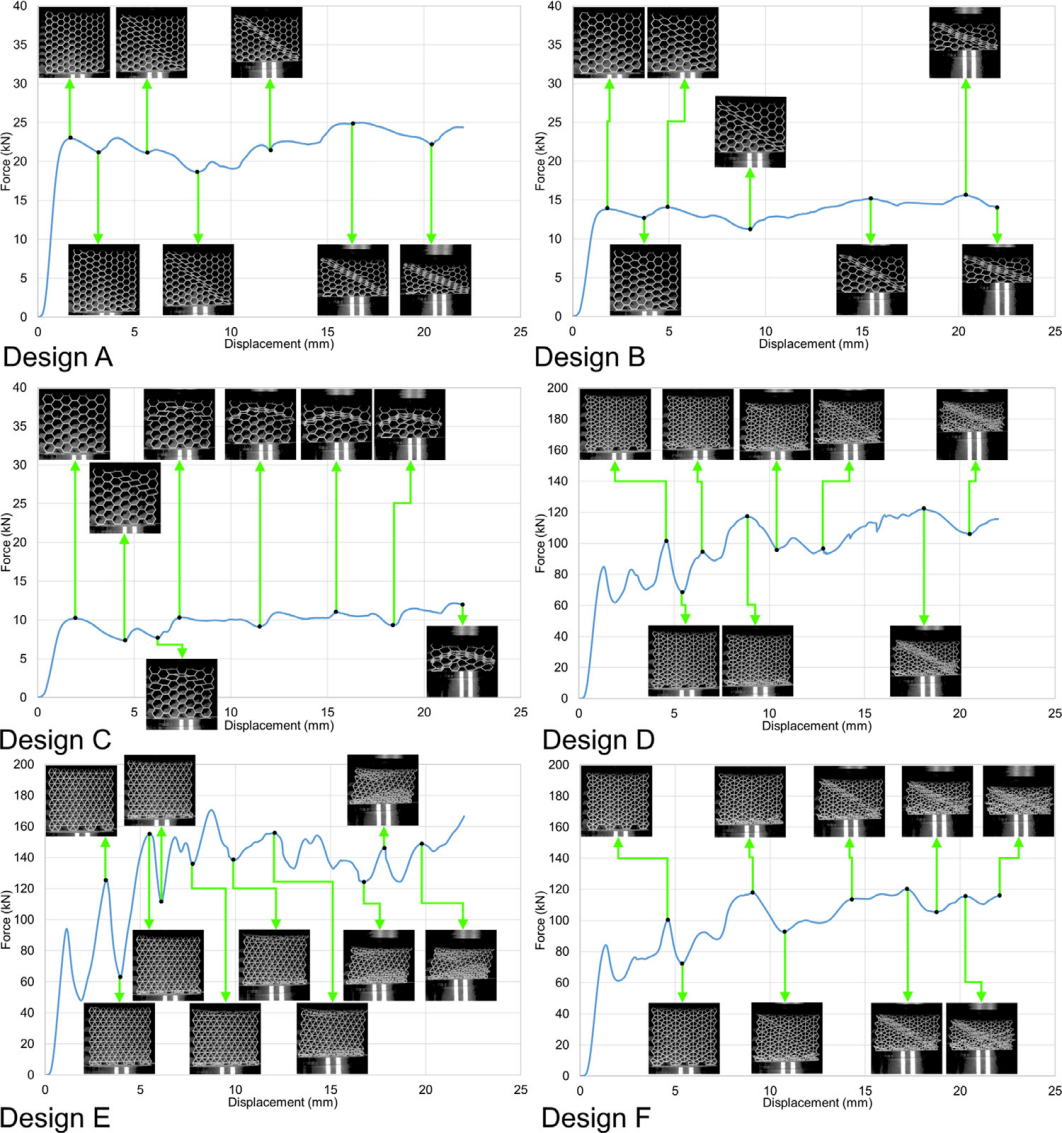
Force-displacement data of the specimens loaded along the Y direction under the deformation rate of 1.8 mm/s.

The load-displacement curves in Figs. 5, 6, 7, and 8 are plotted from the data recorded during the in-plane compression tests (loading along the X or Y direction in Fig. 3) of the specimens as raw files with csv format. The raw data are available in the repository (folder name: In_plane_compressions) and are named following the sample design, loading direction, and displacement rate. For example, A_X_0.1.csv belongs to the sample with design A, loaded along the X direction under the displacement rate of 0.1 mm/s. Finally, the compression tests were also recorded by the DIC system, and the videos are available as mp4 files in the data repository (folder name: In_plane_compressions). The Video files of the compression tests are also named with letters and numbers representing their corresponding sample designs, loading direction, and displacement rate. For example, A_X_0.1.mp4 belongs to the compression test carried out on the sample with design A, loaded along the X direction with the displacement rate of 0.1 mm/s, which corresponds to the curve presented in Fig. 5's top left graph. Snapshots from the videos are also superimposed on the graphs to indicate the test progress following its force-displacement data.

**Table 1:** Surface roughness values from the line scans in Fig. 2 (lines are numbered from top to bottom in the surface images in Fig. 2).

Sample design	Line number	*Ra* (µm)	*Rz* (µm)	Average *Ra* (µm)	Average *Rz* (µm)
A	1	3	22	3	23
A	2	3	28	3	23
A	3	2	17	3	23
A	4	3	32	3	23
A	5	3	16	3	23
A	6	3	23	3	23
A	7	3	24	3	23
A	8	3	30	3	23
A	9	2	16	3	23
A	10	3	22	3	23
A	11	3	21	3	23
A	12	4	32	3	23
A	13	2	19	3	23
A	14	2	19	3	23
A	15	3	20	3	23
A	16	2	17	3	23
A	17	3	24	3	23
A	18	3	20	3	23
A	19	3	34	3	23
A	20	3	23	3	23
A	21	2	16	3	23
B	1	2	14	3	20
B	2	2	15	3	20
B	3	2	19	3	20
B	4	3	20	3	20
B	5	3	26	3	20
B	6	3	25	3	20
B	7	3	28	3	20
B	8	3	21	3	20
B	9	3	24	3	20
B	10	3	24	3	20
B	11	3	29	3	20
B	12	2	14	3	20
B	13	3	26	3	20
B	14	2	14	3	20
B	15	3	17	3	20
B	16	3	25	3	20
B	17	2	16	3	20
B	18	2	18	3	20
B	19	3	20	3	20
B	20	2	19	3	20
B	21	2	14	3	20
C	1	2	20	2	23
C	2	2	15	2	23
C	3	2	18	2	23
C	4	3	27	2	23
C	5	2	15	2	23
C	6	3	34	2	23
C	7	3	25	2	23
C	8	2	11	2	23
C	9	2	20	2	23
C	10	3	24	2	23
C	11	3	30	2	23
C	12	2	22	2	23
C	13	3	33	2	23
C	14	2	18	2	23
C	15	2	19	2	23
C	16	2	26	2	23
C	17	2	22	2	23
C	18	4	22	2	23
C	19	3	31	2	23
C	20	2	21	2	32
C	21	3	23	2	23

**Fig. 9:**
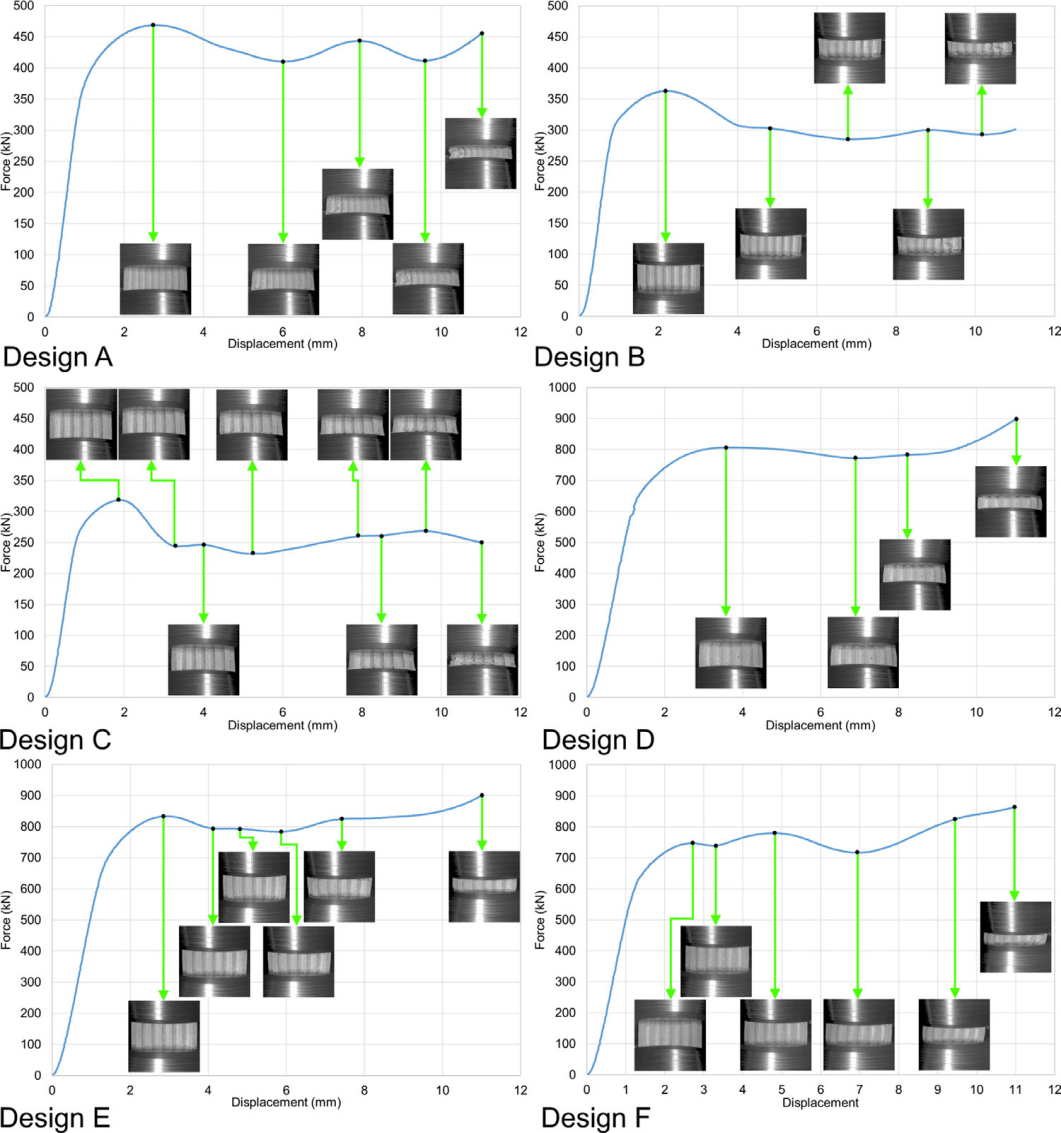
Force-displacement data of the specimens loaded along the Z direction under the deformation rate of 0.1 mm/s.

**Fig. 10:**
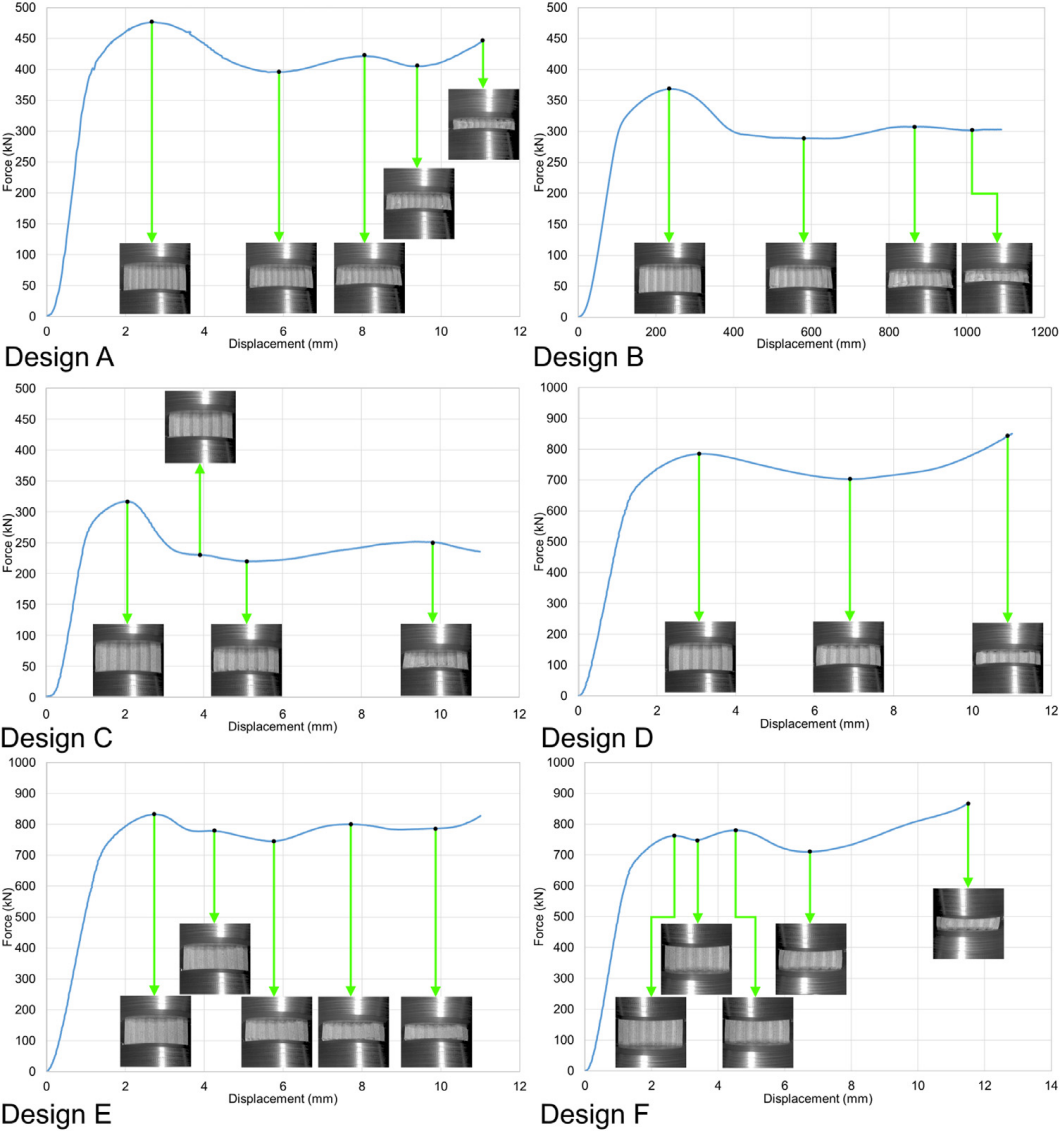
Force-displacement data of the specimens loaded along the Z direction under the deformation rate of 1.8 mm/s.

Similarly, load-displacement curves from the out-of-plane compression tests (loading along the Z direction) of the samples are presented in Figs. 9 and 10. The load-displacement data were recorded and analyzed using the same approach for the in-plane compression tests. The raw files are available in the data repository (folder name: Out_of_plane_compressions) as csv files named per the sample designs, loading direction, and displacement rates. For example, A_Z_0.1.csv belongs to the sample with design A, loaded along the Z direction under the displacement rate of 0.1 mm/s. Similarly, the tests were recorded as video files by the DIC system and are available in the data repository. For example, A_Z_0.1.mp4 shows the test progress of the out-of-plane compression test carried out with the displacement rate of 0.1 mm/s on the sample with design A.

Finally, the impact tests in [1] were visually recorded as high-frame-rate videos. The video files are available in the data repository (folder name: Impact_tests) and are named with codes representing the test type, sample design, and impact load direction. For example, impact_A_X.mp4 belongs to the sample with design A that endured the impact load along its X direction.

## 2 Experimental Design, Materials and Methods

Microstructural analysis was carried out on an additively manufactured cuboid of 10 mm × 10 mm × 10 mm dimensions mounted in epoxy resin. The specimen was ground sequentially with abrasive pads with grit numbers from 400 to 2000; the sample was polished using 1-µm colloidal silica solution. After polishing, the specimen was etched by immersion in Kalling's reagent (1.5 g CuCl_2_ + 33 ml HCl + 33 ml C_2_H_5_OH + 33 ml distilled water) for 15 s, per instructions in [3]. The etched specimen was next washed with ethanol and dried under a stream of cold air. Finally, the prepared specimen was subjected to ultra-sonic cleaning in ethanol for 300 s and then placed inside the chamber of a SU3500 scanning electron microscope. A scanning voltage of 15 kV, an aperture size of 30 µm, the secondary electron detector, and magnifications of 500 and 1000 were used to take the microstructural images.

The tensile test (Fig. 2) was carried out via a Galdabini Quasar 600 universal testing machine in conjunction with an ARAMIS digital image correlation (DIC) system. The test was done under the strain rate of 0.001 s^−1^ at room temperature on a sample with as-built surface quality (not machined or polished) and dimensions under ASTM E08 [2]. Also, the DIC system was calibrated prior to the test following the parameters available in DIC_calibration.pdf from the data repository. The raw data from the DIC system were turned into numerical results and curves via GOM Correlate software (version 2021) using its capability to apply a smoothing spline temporal filter with a degree of 30 on the raw data. Further, the whole course of the tensile test was recorded via the DIC system's cameras with a constant frame rate of 4 fps. Consequently, each time index in Fig. 2 and its related videos represent a 0.250 s time interval.

The surface images and topological features were captured using a KEYENCE VE-3200 3D measuring microscope using 80X magnification. The raw data obtained by the microscope were analyzed and turned into topological data using VR-3000 G2 software version 2.5.0.116.

The in-plane compression tests were carried out at room temperature using an in-house 200 kN servo-hydraulic test rig equipped with a 200 kN load cell from Interface (model 1032AF-225KN-B) to measure the force values; a National Instruments NI-9215 input module was also used to transfer the data into a computer equipped with an in-house user interface developed via LabVIEW to save the results as csv files. The data recorded in the raw files were analyzed and turned into graphical curves using Microsoft office 365’s Excel version 2209. Finally, the compressions tests were recorded by the DIC system's cameras, and the recorded videos were turned into mp4 files using the GOM Correlate software (version 2021). Constant frame rates of 1 fps and 14 fps were used to record the tests with constant displacement rates of 0.1 mm/s and 1.8 mm/s, respectively. Consequently, each time index in the videos represents a 1 s time interval for the tests with 0.1 mm/s rate and a 0.071 s interval for the tests with 1.8 mm/s rate.

Similarly, the out-of-plane compression tests were done at room temperature using an in-house 5000 kN servo-hydraulic test rig equipped with Interface's 1000 kN load cell. The load-displacement data were recorded and analyzed using the same approach used for the in-plane compression tests. Similarly, the tests were recorded as mp4 video files by the DIC system. Constant frame rates of 5 fps and 14 fps were used to record the tests with constant displacement rates of 0.1 mm/s and 1.8 mm/s, respectively. Consequently, each time index in the videos represents a 0.2 s time interval for the tests with 0.1 mm/s rate and a 0.071 s interval for the tests with 1.8 mm/s rate

Finally, the impact (drop) tests were carried out at room temperature using a drop weight and height of 45 kg and 3340 mm, respectively. The tests were visually recorded as high-frame-rate videos via a Phantom VEO 710L camera with a maximum frame rate of 24000 fps. The visual data achieved by the camera were analyzed and transformed into mp4 videos with a play speed of 30 fps using CV 3.5 software version 3.7.802.0; each frame represents a 41.66 µs time interval. Also, each unit on the gauge on the right side of the videos is 1 mm.

## Ethics Statements

This manuscript adheres to ethics in publishing standards.

## CRediT authorship contribution statement

**Shahriar Afkhami:** Conceptualization, Methodology, Data curation, Software, Investigation, Writing – original draft, Writing – review & editing, Visualization. **Mohsen Amraei:** Conceptualization, Methodology, Data curation, Investigation, Writing – review & editing. **Ilkka Poutiainen:** Project administration, Conceptualization, Methodology, Writing – review & editing. **Leroy Gardner:** Writing – review & editing, Validation, Methodology, Investigation. **Heidi Piili:** Writing – review & editing, Resources, Funding acquisition. **M. Ahmer Wadee:** Writing – review & editing, Validation, Methodology, Investigation. **Antti Salminen:** Writing – review & editing, Resources, Funding acquisition. **Timo Björk:** Supervision, Conceptualization, Resources, Methodology, Investigation, Funding acquisition, Writing – review & editing.

## Declaration of Competing Interest

The authors declare that they have no known competing financial interests or personal relationships that could have appeared to influence the work reported in this paper.

## Data Availability

Additively manufactured metal honeycombs (Original data) (Mendeley Data).
